# PCR standard curve quantification in an extensive wastewater surveillance program: results from the Dutch SARS-CoV-2 wastewater surveillance

**DOI:** 10.3389/fpubh.2023.1141494

**Published:** 2023-11-02

**Authors:** Erwin Nagelkerke, Wouter A. Hetebrij, Jaap M. Koelewijn, Jannetje Kooij, Anne-Merel R. van der Drift, Rudolf F. H. J. van der Beek, Eline F. de Jonge, Willemijn J. Lodder

**Affiliations:** Centre for Infectious Disease Control, National Institute for Public Health and the Environment (RIVM), Bilthoven, Netherlands

**Keywords:** WBE, wastewater, sewer, surveillance, standard, calibration, qPCR assay

## Abstract

Since the start of the COVID-19 pandemic in 2020, wastewater surveillance programs were established, or upscaled, in many countries around the world and have proven to be a cost-effective way of monitoring infectious disease pathogens. Many of these programs use RT-qPCR, and quantify the viral concentrations in samples based on standard curves, by including preparations of a reference material with known nucleic acid or virus concentrations in the RT-qPCR analyses. In high-throughput monitoring programs it is possible to combine data from multiple previous runs, circumventing the need for duplication and resulting in decreased costs and prolonged periods during which the reference material is obtained from the same batch. However, over time, systematic shifts in standard curves are likely to occur. This would affect the reliability and usefulness of wastewater surveillance as a whole. We aim to find an optimal combination of standard curve data to compensate for run-to-run measurement variance while ensuring enough flexibility to capture systematic longitudinal shifts. Based on more than 4000 observations obtained with the CDC N1 and N2 assays, taken as a part of the National Sewage Surveillance program at the Dutch National Institute for Public Health and the Environment, we show that seasonal and long-term shifts in RT-qPCR efficiency and sensitivity occur. We find that in our setting, using five days of standard-curve data to quantify, results in the least error prone curve or best approximation. This results in differences up to 100% in quantified viral loads when averaged out over a nationwide program of >300 treatment plants. Results show that combining standard curves from a limited set of runs can be a valid approach to quantification without obscuring the trends in the viral load of interest.

## 1. Introduction

The idea of monitoring pathogens through wastewater has been applied for decades (1, 2), but interest in waste waterbased epidemiology (WBE) has significantly increased with its applications during the COVID-19 pandemic. SARS-CoV-2 detection in wastewater is currently employed as a monitoring tool in over 70 countries, with several programs reaching significant population coverage (3).

Many of these surveillance programs, and WBE studies on SARS-CoV-2 in general, make use of reverse transcription quantitative polymerase chain reaction (RT-qPCR) on one or more regions of the virus genome as the primary method to quantify the number of RNA copies in wastewater samples (4, 5). Absolute quantification of RNA concentrations in this way relies heavily on the use of standard curves with which the initial RT-qPCR results, in the form of cycle threshold values (Ct-values), are converted to a known concentration.

Similar to virtually all measurement instruments, RT-qPCR is known to produce results with test–retest variability. That is, multiple tests of the same sample are known to result in slightly different Ct-values. This not only applies to the results of the Ct-values of samples, but also to the Ct-values of the standard dilutions that result in PCR efficiency estimates and ultimately determine the standard curve that is used for quantification. Although such variation is smaller in (synthetic) preparations with known RNA particle counts, this variation can still affect the quantification process. Therefore, it is recommended to construct a standard curve using multiple replicates and dilutions for each assay (6–8).

The sources of variation in outcomes between duplicate analyses are plentiful and often hard to identify, as it may be any external factor that has the potential to affect the efficiency and sensitivity of the RT-qPCR, as small as changes in mains voltage (9). These may cause stochastic measurement error, where variations occur due to, e.g., randomness in the chemical processes during the RT-qPCR, temperature fluctuations, or external factors such as minor differences in the preparations made by laboratory technicians. Yet, other sources of variation in outcomes, such as the use of different batches of chemicals, seasonal differences in laboratory atmosphere, or general wear and tear of equipment may result in longer-term, systematic changes.

On the one hand, in a more traditional research setting with a predetermined number of experiments conducted in a limited timeframe, such structural external influences are easier to control than in a long-term monitoring setting. When analyses continue over a period of multiple years, it becomes prohibitively difficult to guarantee exactly identical circumstances, chemicals from the same batch run out, and there is personnel turnover. On the other hand, with continuous analyses, the opportunity exists to combine standard curve data from multiple runs, avoiding the need to duplicate the dilutions of the reference material in each run.

This implies that a trade-off exists between on the one hand combining duplicates between runs that reduce random variation caused by stochastic measurement error, and on the other hand updating the standard curve over time to take systematic shifts into account that would bias the quantification process when ignored. This issue of systematic shifts is also identified by Bivins et al. (10), who suggest to monitor shifts in Ct-values over time to determine when to replace the reference material, or use an overall calibration curve based on a mixture model to incorporate run-to-run variability. The trade-off is implicitly recognized throughout the literature where duplication is often recommended, but with consideration for between-run variability (e.g., 8).

Here we investigate this trade-off and present the results from the Dutch National Wastewater Surveillance (NRS) program, based on more than 4000 standard curves obtained using the US CDC SARS-CoV-2 assay targeting two parts of the nucleocapsid protein (N1 and N2 assays) (11), between September 2021 and November 2022. Based on these data we propose and investigate an approach of combining observations of the standard from multiple runs to reduce the effect of random variations in the analyses, while still taking systematic shifts in RT-qPCR efficiency and sensitivity into account. This approach has the potential to be a cost-saving method in high-throughput programs, when it can reduce or eliminate the requirement to duplicate reference material series per run, and may allow quantification of samples from successful PCR runs with an erroneous standard curve. Conversely, in programs with lower analysis frequencies the method may instead lead to a reduction of unwanted variation in standard curve estimation without extra resources, when the results for the reference material can be combined over multiple runs.

## 2. Materials and methods

### 2.1. Standard curve methodology

#### 2.1.1. Log-linear standard curves

Absolute quantification by using a standard curve is an intuitive solution to the problem that initial values are only comparable within one PCR run (12). By including a consistent reference material with, in the case of SARS-CoV-2 analysis, a known RNA concentration the relationship between the Ct-value this reference material produces and its concentration can be determined. By including multiple dilutions of reference material, a curve can be constructed that allows interpolation of the relationship between the Ct-value and the RNA concentration. These standard dilutions are generally 10-fold because of practical considerations in laboratory protocols. Due to the expected exponential growth of particles in the RT-qPCR process, the theoretical relationship between the Ct-value and concentration can be described as:


(1) $$Ct=\mathrm{Intercept}+\mathrm{Slope}*\log_{10}\left(\mathrm{Concentration}\right)$$

Since not the Ct-value is of interest, but the RNA concentration of the sample, this is rewritten as:


(2) $$\log_{10}\left(\mathrm{Concentration}\right)=\left(Ct-\mathrm{Intercept}\right)/\mathrm{Slope}$$

With the increasing popularity of RT-qPCR, advances to the basic approach have been developed, many of them focusing on the implicit assumption that the efficiency is equal for all samples included in a PCR run (13–15). Further improvements focus on better estimates of the efficiency, and a higher precision of the resulting standard curve (16–18). However, despite these efforts, simple ordinary least squares (OLS) curve estimation is still predominant in current research. This may be explained by the intuitive theory behind it, the need to maintain comparability to earlier studies, and the varying results of alternatives (14, 19). Moreover, it is surprisingly rare that obtaining the true RNA or DNA particle count is needed, and in the vast majority of applications maintaining an acceptable level of unbiased comparability between analyses suffices.

Since RT-qPCR is an approximately exponential process whereby PCR doubles the number of DNA particles per cycle, the ideal circumstance would be that each unit increase in Ct-value leads to exactly doubling the number of particles per amplification cycle. On a base-10 logarithmic scale this results in a slope of −3.32. That is, under ideal circumstances, a 3.32 point increase in Ct-value should occur per 10-fold dilution, regardless of the dilution’s absolute particle count.

In that light, the slope of a standard curve is determined by the relative distance between Ct-values of the dilutions of the reference material, assuming that log-linearity holds. As a result, the slope and associated efficiency are primarily indicative of the difference in concentration between high- and low concentration samples or dilutions. Anything that affects the RT-qPCR process in full, is captured in the intercept and could be deemed indicative of the sensitivity.

Of course, directly controllable factors such as the threshold of the analyses linearly affect the resulting Ct-values and can be chosen arbitrarily as long as they are placed in the log-linear phase of the amplification process. However, other factors can impact run-to-run comparability as they may shift the intercept of the standard curve, such as equipment wear and tear, differences between batches of materials and reagents, and laboratory temperatures or humidity. Such factors may affect both the degree of fluorescent luminescence of the sample material, as well as the sensitivity of sensors to the fluorescence (16). When such external factors affect the process equally for all, or a majority, of the dilutions of the reference, the resulting Ct-values change by an approximately equal amount. This would result in a largely unchanged slope, and an increase or decrease of the intercept proportional to the change in Ct-values.

The above has led to suggesting different ways to construct the standard curve when multiple analyses are conducted, based on the circumstances. Generally, when quantifying RNA or DNA from samples, researchers use either the Ct-values of the standard curve per run, or use a master curve where multiple runs are combined. Neither option is very useful or theoretically sensible in long-running surveillance programs, because these would either allow stochastic variance to affect trend estimation, or cause structural changes in quantification parameters to be ignored over time.

Two alternative approaches are based on multilevel random intercept and random slopes mixture models. These approaches combine information from all runs to reduce stochastic error, but allow either the intercept to vary with a fixed slope, or allow both the intercept and slope to vary per run (16). Although elegant solutions, there are some caveats when applying them for long-term, real-time monitoring. Firstly, these methods are introduced with the assumption that the complete data is available before standard curve estimation, and would require extensive computation after each PCR analysis in a continuous monitoring setting. Secondly, and more problematic, is that the approaches do not guarantee that shifts over time are properly captured. The estimation assumes that between-run variation randomly fluctuates around a midpoint. That midpoint, however, suffers the same problem as a master curve and is slow to incorporate systematic shifts due to it being based on all historically available data.

#### 2.1.2. Rolling window master curve

To overcome the latter point, we here suggest an approach that uses historic data on standard curves within a rolling window as a pragmatic way to take advantage of the continuous observations of the standard in a monitoring setting. Such an approach may simultaneously reduce unwanted, stochastic variance by using more of the available data while still being able to incorporate longitudinal shifts in RT-qPCR efficiency and sensitivity. Due to its widespread use we do so using common standard curve methodology, where future steps are to combine these findings with advances such as using mixture models in curve estimation to allow plate-specific variance in efficiency estimation.

The assumption here is that, on average, an ideal amount of historic data of the reference dilutions exists. Using the data from this period reduces stochastic error in standard curves more than error that is introduced by structural shifts in the standard curves. Specifically, the distance between the expected Ct-values based on the current standard curve, and the Ct-values from the standard curve based on earlier observations of the reference material can be minimized. At the point of the smallest error, the standard curve based exclusively on historic data is the best approximation of the current standard curve parameters. That is, we propose an analysis using previously obtained standard curves, or measurements of the reference dilutions, whereby these observations of dilution series are used to obtain a standard curve that is compared to the current curve.

Because systematic changes are a function of time, the curves based on previous observations of the reference material are here obtained per day, but the span could be any theoretical sensible timeframe, or can be a number of previous runs. Based on the observations within this historical span a standard curve can be constructed using the observed Ct-values of the dilution series. Subsequently, the root mean square error (RMSE) between the curve based on a given number of previous days and the per-run curves of today can then obtained by computing the Ct-values associated with the known particle counts on the line per run, and the difference to the Ct-values resulting from the line based on historical data. Note that the error should be obtained per current day run, and only subsequently be aggregated. Not doing so would average out any differences between standard curves before obtaining the residual, which would significantly reduce the potential impact of individual runs. Doing the above for different amounts of historical data, e.g., 1 through 20 days prior to today, the span that on average best approximates all standard curve observations of today can be determined by minimizing the RMSE. The expectation here is that the ideal tradeoff between systematic and random error can be determined. As more data is used, stochastic error variance will be reduced, but error due to systematic shifts in the standard curve parameters will increase. At the point with the lowest RMSE the reduction in stochastic variance is smaller than the error introduced due to systematic shift in standard curves, identifying the ideal size of the window of the smoothed, rolling window curve.

Further note that the curves estimated on data obtained in the preceding days should be based exclusively on data preceding the current day or current run. Including observations from the current run or day in this analysis would give an arbitrary advantage to curves based on less historic data, since current observations are the best approximation of themselves and would form a larger share of the data when less previous information is used. For this same reason the most current data does need to be incorporated for the final curve that is used for quantification.

As a final remark, in the presented situation, curves based on one or two previous days are systematically missing due to weekends and national holidays. This is resolved by using the last estimate available in the cases where no data is observed on days prior to today. This would be the most pragmatic solution when applying the idea of a rolling window in practice.

### 2.2. Reference material

A synthetic DNA construct containing complementary sequences of the CDC 2019-nCoV Real-Time RT-PCR Diagnostic Panel, consisting of primers and probes that target the nucleocapsid (N) gene (11, G-block sequence in S1), downstream of a T7 RNA-polymerase promotor (Thermo Fisher Scientific) was used to transcribe RNA using the MEGAshortscript™ T7 Transcription Kit (Thermo Fisher Scientific).

Following transcription, and after a DNase step to remove the synthetic DNA construct, the generated RNA was quantified using a clinical isolate with a known concentration of SARS-CoV-2 genome copy numbers. In RT-qPCR, each assay, consisting of different primers/probes, has a different priming reaction. Therefore the performance of the N1 and N2 assay on the generated standard RNA has to be evaluated separately (11).

Ten-fold serial dilutions of the generated standard RNA were tested to determine which dilutions could be included in the quantification curve, resulting in five dilutions of the RNA standard generating a positive signal.

The generated standard RNA was aliquoted in large batches of 7 μL per tube (for single use) and stored in a −80°C freezer. Before each RT-qPCR run the standard RNA is serially diluted for direct use. Per 96-wells PCR plate the RNA of 20 samples are tested in duplicate using the N1 and N2 assays. The RT-qPCR is performed as prescribed previously (11) with minor modifications; Each reaction contained 1x TaqMan Fast Virus 1-step Master Mix (Thermo Fischer Scientific) and a final concentration of 0.5 μM and 0.25 μM of primers and probes, respectively. In each RT-qPCR run, for each assay, a negative control, a positive control and five dilutions of the standard RNA are included. All analyses were performed in an in-house laboratory at the Dutch National Institute for Public Health and the Environment, using nine different QIAquant 96 5-plex instruments (Qiagen). The threshold with which a Ct-value is determined is fixed across all runs in the log-linear phase of the RT-qPCR process.

### 2.3. Data

Data collection took place between September 1st 2021 and November 11th 2022 as part of the Dutch NRS program. During this period approximately 125 to 250 wastewater samples were quantified daily, resulting in four to ten PCR runs on average. In each run, five 10-fold dilutions of the RNA standard were included. However, the most diluted reference resulted in inconsistent Ct-values that strongly affect estimated standard curves. Therefore, only the other four standard dilutions are used for the analyses below. To improve readability of figures these are referred to as −04 to −07 in the following. Although general recommendations for dilution series include 5–6 points, we have confidence that the obtained standard curves are adequate, as they very closely match the curves reported in Bivins et al. (10) for the N1 and N2 CDC assay. Furthermore, if additional 10-fold dilutions were added these can occasionally fall outside the log-linear phase of the quantification curves.

The procedures for the quantification are based on the NEN-EN-ISO-15216-1 (20) standard for hepatitis A virus and norovirus quantification in food chain microbiology. The construction of standard curves prescribed follow the widely used criteria for standard curve estimation, requiring a minimum of three 10-fold dilutions. These should log-linearly result in a slope between −3.60 and −3.10, which equates to a PCR efficiency estimate of 90 to 110%, and a minimum correlation between standard dilutions of 0.99, which translates to an R-squared of 0.980 or higher. In the assessment of RT-qPCR data obvious deviations in the results of the reference dilutions are manually removed before standard curve construction. An example would be two wells with reference material resulting in almost identical Ct-values.

The ISO standard further allows the removal of dilutions based on outlying Ct-values, maintaining the minimum requirement of three or more observed dilutions per standard curve. Here the following procedure is applied: a standard curve is estimated on all four directly observed dilutions, or three in the case of aforementioned reasons for removal. When the resulting curve does not adhere to the criteria, but an acceptable curve exists when excluding any one out of the four dilutions, we use the combination excluding the most diluted reference that leads to an acceptable curve.

Standard curves were obtained on 308 out of 437 days. All days without data are weekends or national holidays. A total of 2282 unique runs were performed, the majority of which contain two series of RNA standard for both the N1 and N2 gene, respectively resulting in 4455 and 4442 approved dilution series. Applying curve construction as described, this leads to 3755 (84%) and 3145 (71%) curves that adhere to the criteria.

To approximate the comparison between using historical data and the recommended duplication of dilution series of the standard, one line per RT-qPCR duplication is constructed for the results in section 3.3. As mentioned, samples within the NRS program are analyzed in duplicate for both N1 and N2 genes, where 40 wells are used for N1 and 40 wells for N2 on a 96-wells plate. Both runs contain a dilution series. The between-plate dependence of these analyses is high, plates contain identical reference material, and although two separate instruments are often used, the external circumstances are close to identical. These two sets of dilutions from the duplicate runs are combined to better approach the situation in which reference material is duplicated, and a standard curve is estimated on 3 – in the worst case scenario where one of the standard curves is unusable – to 8 dilutions, after which the same criteria as before are applied.

## 3. Results

### 3.1. Univariate descriptive statistics

To be able to combine the generated data and give a single overview, dependencies in the performance of the different qPCR instruments (QIAquants) are tested by conducting an ANOVA on the slopes and intercepts of constructed standard curves. A description and the results of this analysis can be found in S2. The largest mean difference in standard curve intercepts between two machines equals 0.157 for the N1 assay, and 0.220 on the N2 assay. Explained variance in the estimated intercepts by differences in qPCR instruments does not exceed 0.43% for N1 (*F*(8, 3634) = 1.96, *p* = 0.048), and 1.12% for N2 (*F*(8, 2985) = 4.24, *p* < 0.001). The statistical significance is a result of the large sample sizes, whereas the practical implication of these differences is negligible. With regard to the slopes, the mean differences and explained variance are, respectively, 0.024 and 0.38% for N1, and 0.048 and 0.85% for N2. Although this does not exclude temporary, larger differences between pairs of instruments, the results give confidence that the qPCR results from instruments can be combined.

**Figure 1:**
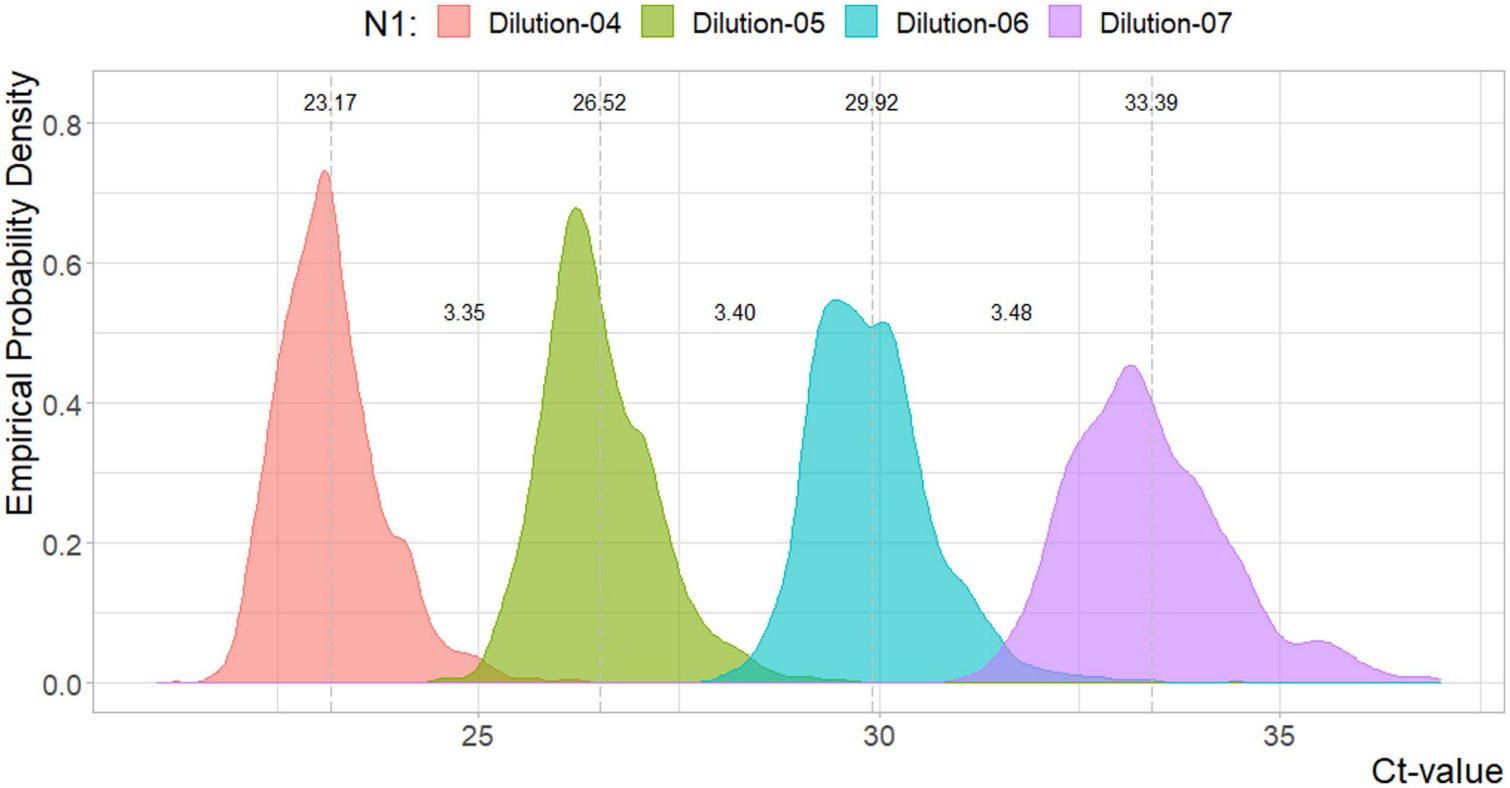
Density plot for the Ct-values per N1 reference dilutions. Dotted lines and values indicate the mean value, and distances between the mean values. Sample sizes are 3754, 3753, 3754, 3689, respectively.

**Figure 2:**
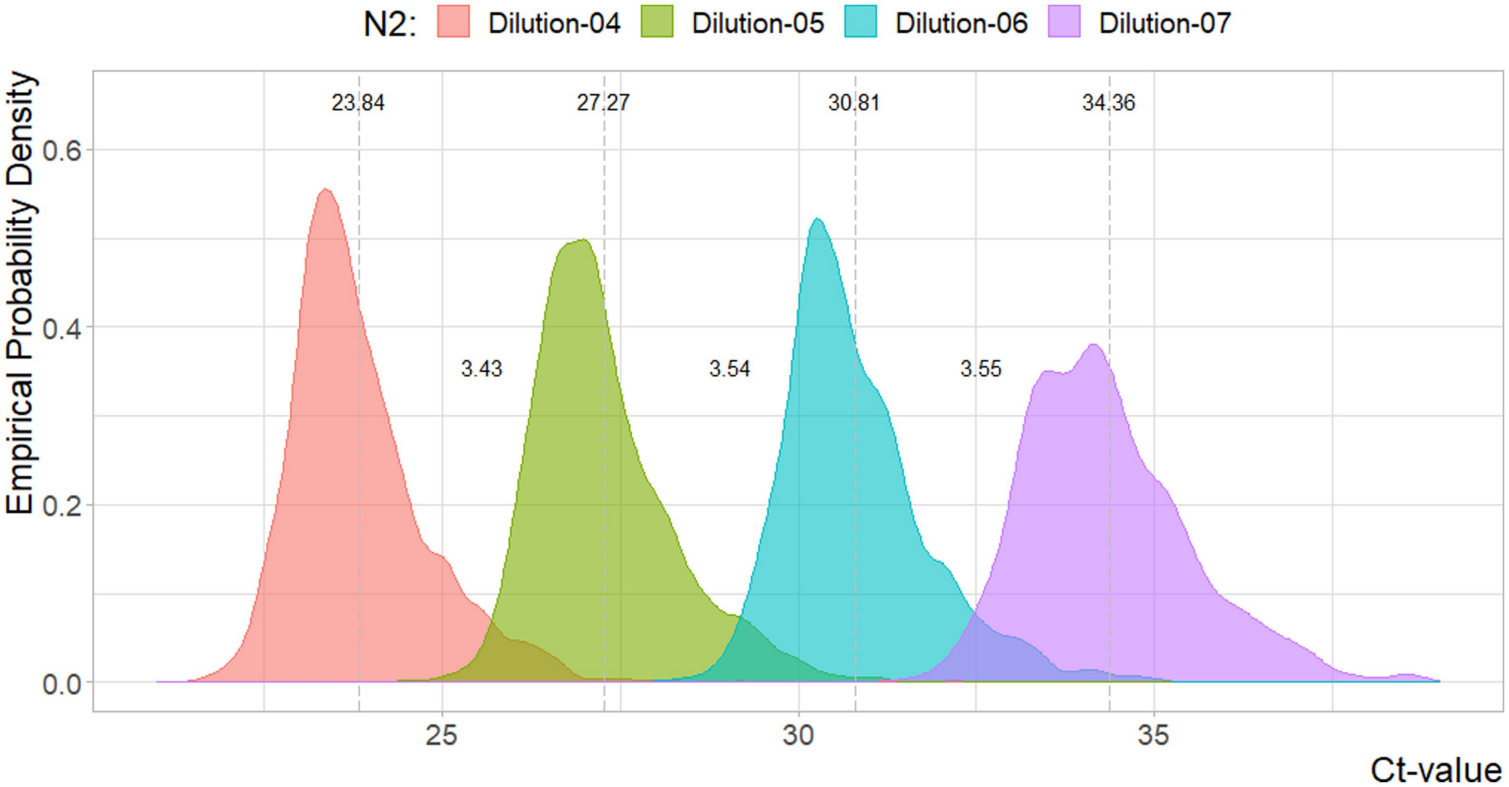
Density plot for the Ct-values per N2 reference dilutions. Dotted lines and values indicate the mean value, and distances between the mean values. Sample sizes are 3144, 3140, 3144, 3049, respectively.

**Figure 3:**
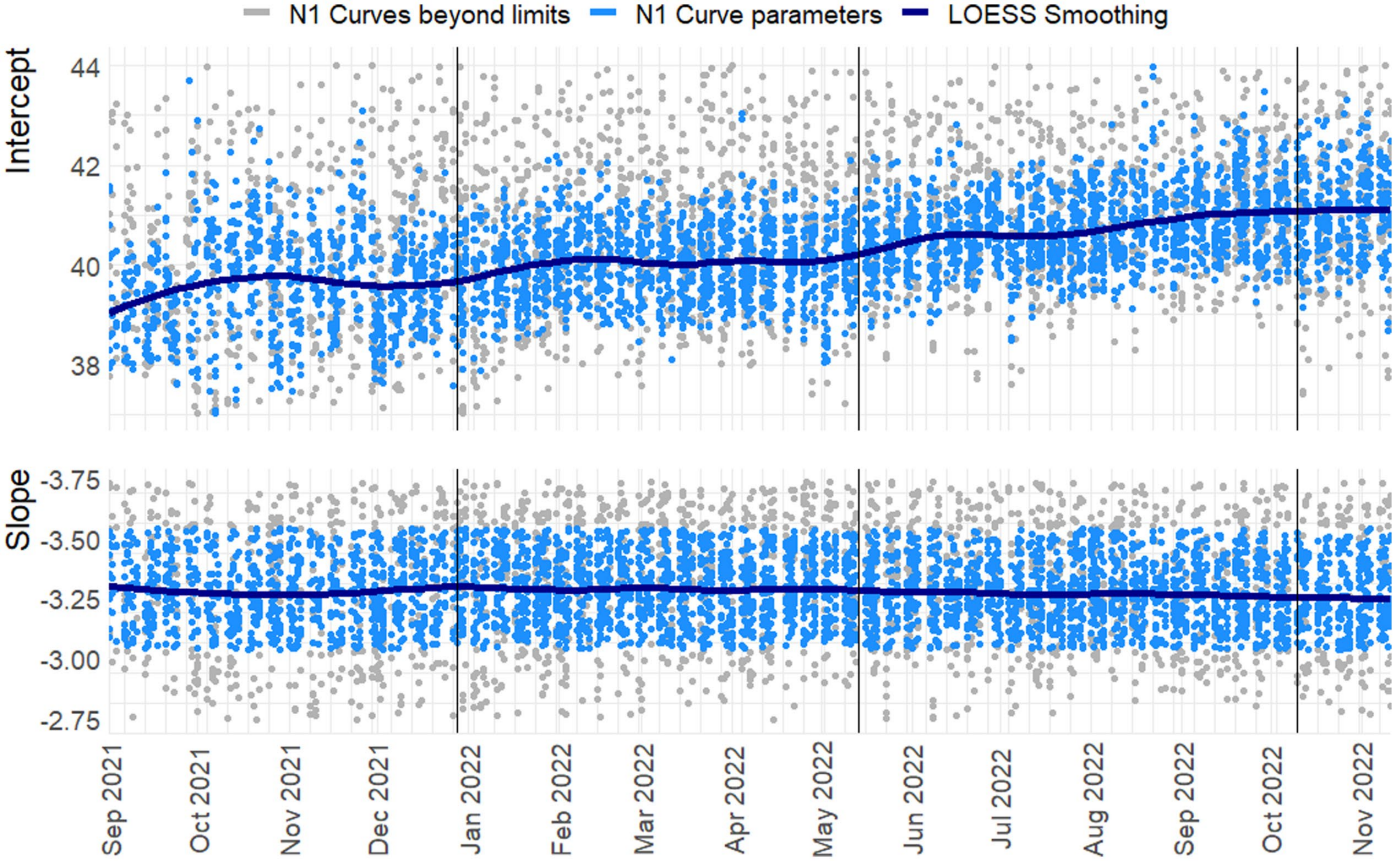
Slope and intercept for the N1 Target. loess smoothing with span = 0.25, degree = 2. N = 3755/4455. Vertical lines indicate a new preparation from the CDC assay. Blue dots indicate line parameters of accepted standard curves, gray dots show rejected curves. For readability 242 and 650 observations are beyond the y-axis limits for the intercept and slope, respectively.

**Figure 4:**
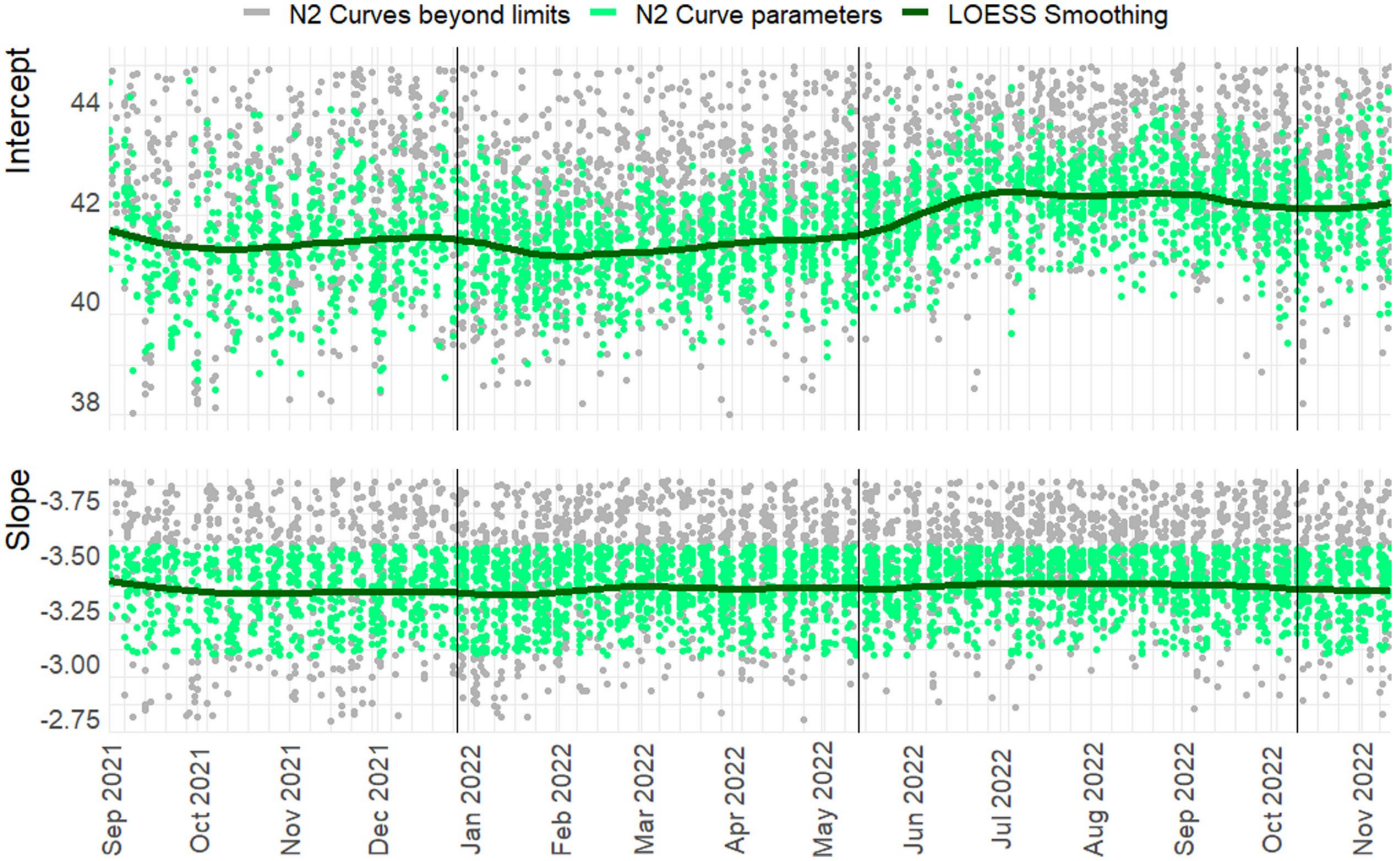
Slope and intercept for the N2 Target. loess smoothing with span = 0.25, degree = 2. N = 3145/4442. Vertical lines indicate a new preparation from the CDC assay. Green dots indicate line parameters of accepted standard curves, gray dots show rejected curves. *Note:* For readability 548 and 855 observations are beyond the y-axis limits for the intercept and slope, respectively.

In Figures 1, 2, the density estimates and mean Ct-values for all observations of the four standard dilutions are shown when any combination leads to a curve within the criteria. Summary statistics are presented in Table 1. For both N1 and N2 the different concentrations show very comparable distributions. Combined with Figures 3, 4, which show a relatively stable long-term slope, the different dilutions seem to be affected in a similar fashion over time. This also explains the slight right-tail skew of the distribution as the intercept is, on average, trending upwards over time. The decreasing kurtosis of the distributions indicates larger variability in lower concentrations, which is expected given the higher levels of uncertainty in PCR analyses in more diluted samples.

**Table 1:** Summary statistics of the reference dilutions.

	N1				N2			
	Mean	Median	Density	Var	Mean	Median	Density	Var
St-04	23.17 (−)	23.08 (−)	23.09 (−)	0.50	23.87 (−)	23.64 (−)	23.37 (−)	0.90
St-05	26.52 (3.35)	26.39 (3.31)	26.21 (3.12)	0.61	27.27 (3.43)	27.08 (3.44)	26.98 (3.61)	1.03
St-06	29.92 (3.40)	29.83 (3.44)	29.46 (3.25)	0.61	30.81 (3.54)	30.60 (3.52)	30.28 (3.31)	1.12
St-07	33.39 (3.48)	33.25 (3.42)	33.13 (3.67)	1.13	34.36 (3.55)	34.18 (3.58)	34.15 (3.87)	1.50

Distances between dilutions between parentheses.

### 3.2. Temporal trends in curve parameters

In Figures 3, 4 the curve parameters estimated on each set of reference material are shown, where the colored scatter indicates standard curves that fall within the selection criteria described in section 2.3. Locally estimated scatterplot smoothing (loess) is used for trend estimation.^1^ Long-term, structural fluctuation in the standard curves, as well as significant run-to-run variation in both the slope and intercept terms are visible. The former, structural trend, mostly manifests itself in terms of the intercept, which implies that all dilutions of the standard are similarly affected by the external factors that cause the shift, and the standard curve as a whole shifts upwards or downwards over time, in line with Figures 1, 2. Shifts in both the N1 and N2 curves are substantial, with approximately 2 Ct difference between the highest and lowest points. A difference of this magnitude will have a notable impact on the virus concentration obtained for the samples. The slopes of the standard curves, and associated efficiency of the analyses, show less structural fluctuation, indicating that the sensitivity of analyses varies more strongly and systematically than their efficiency.

Moreover, the trends in the N1 and N2 intercepts deviate from one another, and often show shifts in opposite directions, while the RNA is generated using the same G-block. For example, in the period from January to February 2022 the N1 assay shows a decrease in sensitivity (increasing intercept Ct-value) and the N2 assay shows an almost equal increase in sensitivity. This is plausible given that the N-gene primers and probes show different concentrations after preparation, but also implies that they react to different external factors, or react differently to similar external factors.

Disregarding the seasonal variation in Figures 3, 4, both assays also show a steady upward trend over the year. This is less apparent for the N2 assay from the figure, due to the trendline being somewhat distorted at the endpoints. The first and last week of data have above and below average intercept estimates, respectively. This upward trend is seemingly small (N1; B = 0.004, t = 39.72, *p < 0.001.* N2; B = 0.003, t = 24.65, *p < 0.001*), but results in a theoretical shift of 1.57 Ct for N1 and 1.18 Ct for N2 over the course of one year, which will cause a significant upward trend in terms of the obtained viral concentrations from samples. We assume this to be the result of general wear and tear, as all data is obtained from PCR equipment that was brand new at the start of the measurements when sensitivity is expected to decline faster during a period of breaking in the new instruments. A similar trend is not observed in data before September 2021 obtained in a laboratory with older equipment.

The slopes in the second panel of the figures indicate good efficiency, but structurally result in efficiency estimates just below the 90% limit. The net result of this is hard to determine. A steeper slope causes smaller differences in concentration for equal differences in Ct-values. However, this may cause both over- and underestimation, and is further compounded by the interaction between the slope and the intercept terms. One explanation may be so-called compound errors; the different standard RNA dilutions are obtained through serial dilution of one batch preparation. In practice it is almost always the case that when minor variations occur, too little standard material is pipetted, causing the assumed concentration to be higher than the true concentration. This error is then carried over to any dilutions made afterwards.

**Figure 5:**
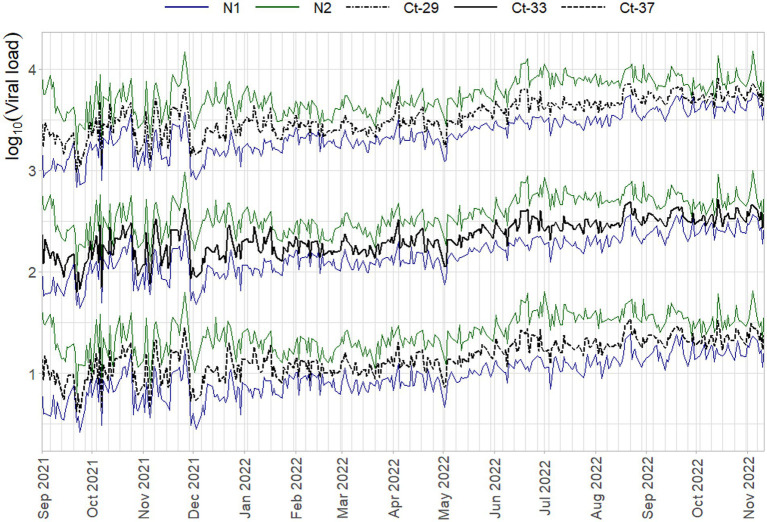
Average daily log_10_ concentration of three Ct-values when quantified with the standard curves per run on that day. N1 (blue) and N2 (green) trends are superimposed on all three Ct-value sets (black lines), which are plotted on a base-10 log-scale to be able to distinguish them from each other.

To inspect if the mentioned interaction between slope and intercept terms does not result in systematic bias, the log_10_ viral load of three Ct-values associated with high (29) / medium (33) / low (37) viral concentrations is plotted over time in Figure 5. Here any systematic over- or underestimation of samples with different concentrations would result in diverging trends over time (i.e., increasing or decreasing distances between the respective log_10_ concentrations), of which there is no evidence. In addition, the N1 and N2 gene show the same overall trend, but have almost diametrically opposed short-term fluctuations, again indicating that external factors affect the sensitivity, efficiency, and quantification of the samples differently.

### 3.3. Rolling window master curve

**Figure 6:**
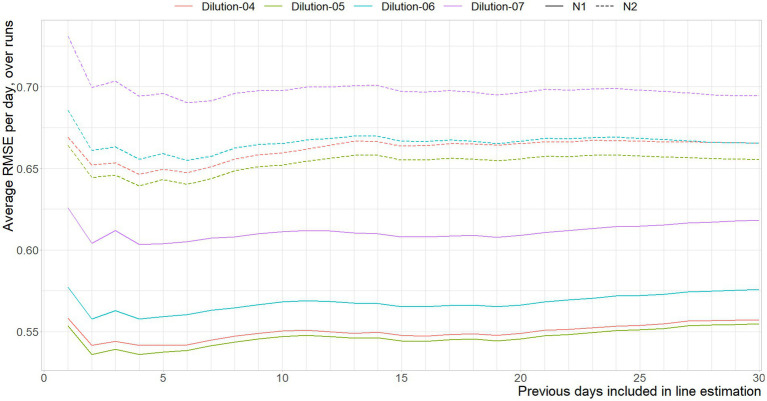
Mean RMSE between the Ct-values interpolated from the standard curve per run, and the standard curve based on *x* previous days, per dilution, per gene target.

In Figure 6 the RMSE between the Ct-values associated with the different dilutions of the reference material are shown, per number of previous days included in the curve based on historic data.

In line with the expectation, including more data to reduce random variance works for a brief period of time, signified by the error reduction as a result of using data over one through four days. The shifts in error reduction on day three for the N2, and between days six and fifteen of the N1 assay can both be explained by the remaining effects of days without observations. The average age of the data used to approximate the current curve fluctuates, and is oldest when Monday is predicted using last week’s data. That is, due to having no new data in the weekend, a prediction based on three days of data causes a prediction of Monday by only using Friday, which explains the sudden increase of error for the N2 assay. This effect re-occurs every seven days, where there is a transition between having either more data available, and that data being older data on average.

**Figure 7:**
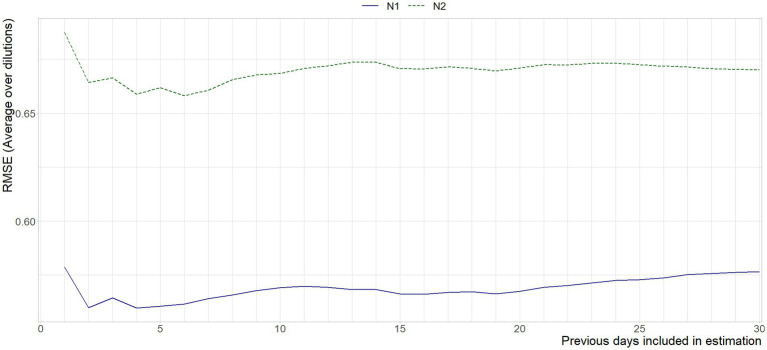
Mean RMSE between the Ct-values interpolated from the standard curve per run, and the standard curve based on *x* previous days, per gene target.

Figure 7 shows the aggregated error between the historic curve and the curves obtained on the current day. The weekend-weekday effect is amplified here, as the error is combined across dilutions. It is nonetheless apparent that using standard curves estimated on the data of four days prior gives the best approximation of today’s standard curves, although it should be noted that using between four and seven days would not give significantly different results, both in the statistical and substantive sense.

**Figure 8:**
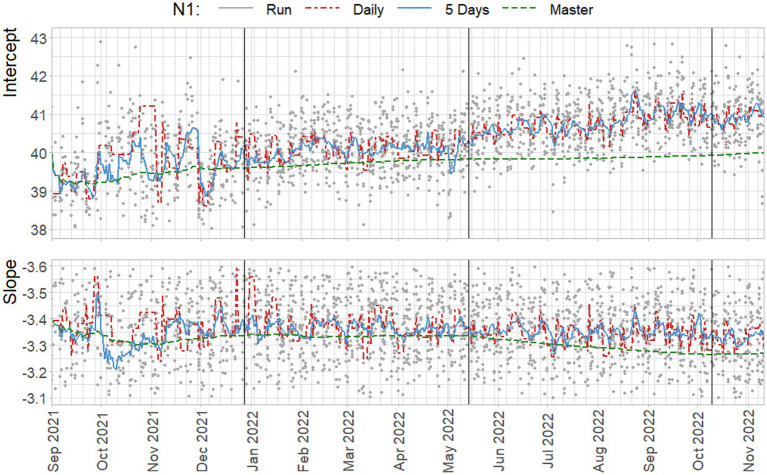
Estimated standard parameters for the N1 target, based on estimation per run (gray), on all daily observations of standard dilutions (red), on all observations within a five day rolling window (blue) and on all cumulative data up to that point (green). Vertical lines indicate a new preparation from the CDC assay.

Figure 8 contains the slopes and intercepts for three methods – duplicate standard combined in two simultaneous runs, a cumulative master curve using all data up to that point, and the suggest rolling curve over five days of data – when used to estimate standard curves using the Ct-values of the N1 standard dilutions (see S3 for N2). To depict the trend in the day-to-day quantification, a line is also estimated using the combined data from each day. As previously mentioned, the rolling curve was re-estimated to include a total of five days: the four previous dates as shown in Figure 6, plus today.

The suggested approach of including five days of data results in standard curves that follow the trend in day-to-day variation, with the exception of October 2021, and the third week of August 2022. In contrast, the master curve clearly shows increasing underestimation of the intercept and steepness of the slope compared to the other methods. Note that the slope is negative, and a shallower, less negative, slope indicates increased efficiency. This result is, of course, specific to the trends in standard curve parameters that are observed in the data from the NRS program, where a steady increase in intercepts, and decrease in slopes occur in the data.

Whether or not over- or underestimation of the concentration occurs depends on the observed Ct-value of a sample, and the over- or underestimation of the intercept and slope. For example, when a high intercept and steep slope are found, it may be the case that the concentration of samples with low Ct-values may be underestimated based on the high number of cycles associated with the intercept. However, due to the steep slope, using the same curve may cause overestimation of the concentration of samples with high Ct-values.

### 3.4. Trends in quantified viral concentrations

**Figure 9:**
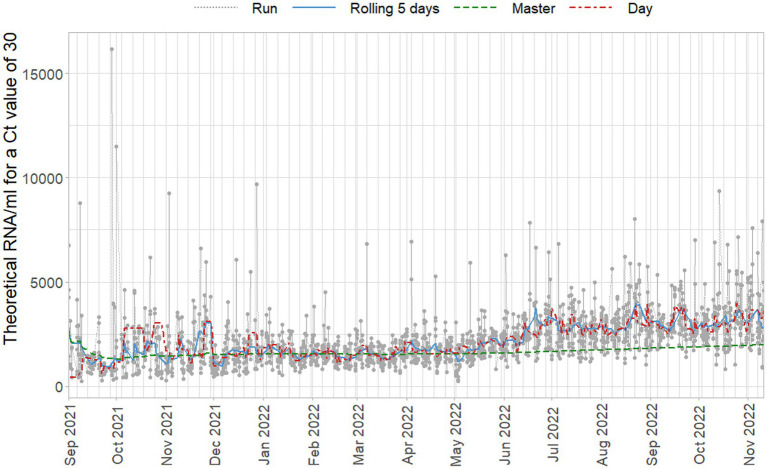
Estimated concentrations for a fixed Ct-value of 30, based on standard curves per duplicate run (gray), per day (red), on a five day rolling window (blue), and cumulative master curve (green).

In Figure 9 a fixed Ct-value of 30 is quantified using the different standard curves, of which the parameters are plotted in Figure 8. Note that general trends occur gradually even when new preparations of the standard are made, and these do not seem to cause sudden changes.

Figure 9 makes clear how relatively limited changes in the standard curve parameters can have large effects on the sample concentrations due to the logarithmic relationship between Ct-values and concentration. Even when applying replication of the standard dilutions, run-to-run variation can be high between days and regularly causes extreme values. Standard curves of these runs adhere to the specified criteria and although their intercept terms, for which no criterion is applied, are relatively high, they are not so high as to raise immediate suspicion in light of the daily variation observed. Concentrations of that magnitude would, of course, be subject to further inspection after quantification as clear outliers, but the properties of the standard curve do not indicate problems in isolation.

The proposed approach of including several previous days reduces the run-to-run, and day-to-day variance. Due to the outlying virus concentrations based on per-run standard curves, Figure 9 does not do full justice to the day-to-day variance. For example, in December 2021 there is an almost 100% larger shift in viral concentration based on daily standard curves than based on the other approaches to quantification. Moreover, daily runs already partially use historical data due to the use of the ISO criteria for the curves and using the most recently accepted curve if the current curve is problematic. This paradoxically leads to the situation where the daily standard curves capture less of the variance in standard curve variance, and would occasionally show almost equally extreme concentrations as run-based curves when they are used on face value.

**Figure 10:**
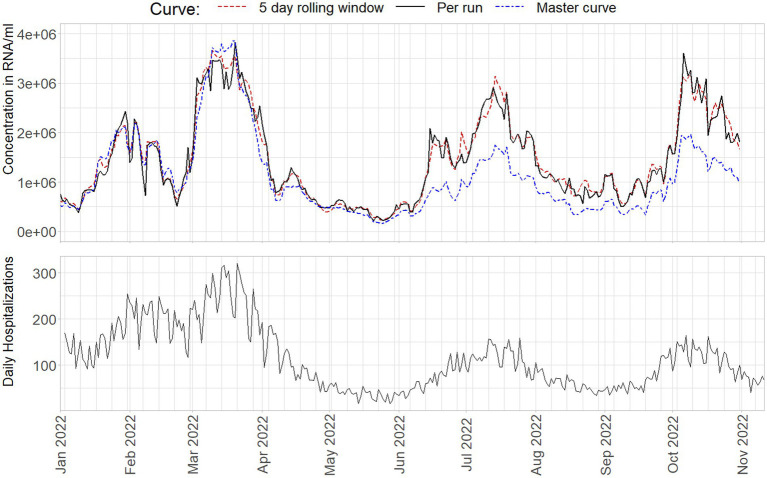
National level aggregate virus loads from January 1st 2022 to November 11th 2022 as obtained from a standard curve per run (black), on a 5-day rolling window (red), or a cumulative master curve (blue), compared to daily hospitalizations.

Finally, in Figure 10 a selection of 44500 samples from the NRS program, collected between January 1^st^ and November 11^th^ 2022 are quantified and aggregated to country level using the described methods. For details on sample collection, aggregation, and hospitalization data, see Geubbels et al. (21). Samples are only included if a standard curve is available for the RT-qPCR run on which they were originally analyzed. Note that due to data selection, and not using inflow or inhabitant corrections for the final concentration, the trend presented cannot be interpreted as the viral trend in The Netherlands. It does nonetheless track the virus concentration to an acceptable level for the purpose of this study.

The master curve starts of performing very similar to the 5 day rolling window standard curves. However, as can also be seen from Figures 3, 4, 8, increasingly underestimate the virus concentrations over time compared to the other methods, where the signal is decreased to about 50% of the virus loads compared to the alternative approaches. Per run curves show more, and especially in the beginning of 2022, unrealistic levels of variance, with changes in estimated virus concentrations occasionally in- or decreasing more than 100% between days.

Differences between the rolling and run-to-run quantification approaches can exceed 400%, but these cases are an exception where the standard curve of one run is markedly different from the rolling window curve. The median absolute difference on the daily nationwide average equals 8%, Some noticeable, prolonged periods of larger differences, upwards of 25% between the approaches, can be seen in the first and third peak in the data. The median absolute difference on the daily nationwide average equals 8%, with prolonged period in the first and third peak in the data showing differences upwards of 25%. The difference between the reported median percentages also illustrates that on a higher level of aggregation the effect of a different standard curve will be smaller, as the viral loads averages out over many observations. On more regional, or on WWTP level, trend estimates will show more marked differences.

This is far from being definitive proof, but when comparing the virus concentrations to the number of daily hospitalizations as a proxy variable, the standard curves using several previous days of data seem to outperform the other methods in capturing the correct levels of virus concentration on the aggregate level. Note that the lower peaks in hospitalizations do not indicate better performance of the master curve, as immunity through vaccination changed the ratio between viral load and hospitalizations over time (22, 23). This is especially important toward the end of 2022, as the standard curve affects estimated trends more severely in high virus concentrations: Small shifts in the curve intercept and slope have the potential to cause large trend variations by the nature of Ct-values being logarithmically related to virus concentration.

## 4. Conclusion

We present the resulting standard curves from more than 4000 analyses of standard dilutions constructed from the CDC 2019-nCoV Real-Time RT-PCR Diagnostic Panel over the period of sixteen months. Further we propose a method of combining observations of standard dilutions over multiple runs in a high-throughput wastewater monitoring setting.

Results indicate that both the slope and intercept parameters of standard curves show high levels of run-to-run variance, and are subject to systematic shifts over a year that can exceed 2 Ct-values. Especially the sensitivity of the RT-qPCR process varies, as indicated by changes in the intercept of the standard curves. This is an important finding with regards to commonly applied criteria for standard curve construction, as the intercept is often, and surprisingly, absent from the quality indicators. As a result it may be a parameter that receives less attention when efficiency estimates are within their bounds, despite the potentially large impact on the concentrations obtained from samples.

The systematic fluctuations in the estimated standard curves make the application of a general master curve problematic. As time progresses the amount of data required to shift a master curve toward its current true value becomes exceedingly large, until the curve is virtually constant. In contrast, concentrations obtained from standard curves obtained from individual dilution series, even when duplicated on simultaneous runs, can result in large variation, with obtained sample concentrations that can exceed surrounding measurements by an order of magnitude.

Using the proposed approach of combining standard curve data from multiple runs, may not only have the practical benefits of extending the period for which the reference batch is identical, and cost-saving through less wells per run being occupied by reference dilutions. Results indicate that it also has the potential to solve problems associated with standard curve estimation, as a more plausible degree of variation in curve parameters is obtained than from run based standard curves, while systematic changes are properly captured. Both of these can be beneficial in high throughput surveillance programs, such as wastewater surveillance.

The newest data is often of major importance to fulfill the real-time monitoring objective that many surveillance programs are tasked with. Problematic or outlying concentrations can compromise this task, given potential delays through re-testing or partial rejection of output. Depending on the degree to which true variance is captured by partially smoothed standard curves, the proposed approach can reduce such problematic data where they are caused by standard curves that contain significant measurement error. Furthermore, trends in the data roughly coincide with meteorological seasons. Although multiple years of data would be required and controlled for laboratory atmosphere to ascertain this with certainty, if these trends do indeed have a yearly recurrence they may cause the viral load signal of interest to be dampened or amplified. Specifically during autumn and winter seasons there is a potential for these trends to coincide with seasonal variations of certain infectious disease prevalence, most notably respiratory viruses such as influenza and SARS-CoV-2.

Despite promising results, this work should be seen primarily as a proof of concept, since the major caveat is that the true standard curve is unknown. Given the observed changes in qPCR efficiency and sensitivity, and the theoretical basis for standard curve estimation, usage of a master curve for a longer period of time can be advised against. However, whether short term variance is captured to a sufficient degree when using standard curve estimation on a rolling window requires further work through a simulation study in which the properties of the true standard curves are known, and recovery of those curves can be investigated.

Such a study would further allow investigation of the required density of standard curve data. Preliminary results obtained over the year prior, where the weekly analysis frequency was one fourth of the data presented, show similar results. However, whether or not good approximation of standard curves per run is possible by using a rolling window, is dependent on the specific properties of the RNA or DNA target, the reference material used, and the laboratory setup and equipment, combined with data availability over a given period of time.

## Data availability statement

The datasets presented in this article are not readily available because contractual obligations with third parties providing data to the program prohibit sharing the data contained in this manuscript without reservation. Full analysis results, programming code, and underlying data are available upon request. Requests to access the datasets should be directed to afvalwatersurveillance@rivm.nl.

## Members of consortium

National Wastewater Surveillance Program (NRS), National Institute for Public Health and the Environment (RIVM), Bilthoven, Netherlands.

## Author contributions

EN: conceived and wrote the manuscript, conceived and implemented the method described therein. WL: designed and set up the infrastructure and laboratory methods of the Dutch National Sewage Surveillance program, wrote parts of the manuscript related to laboratory methods, and contributed to manuscript revisions. JMK: constructed reference materials, assisted in data collection, and reviewed the final manuscript. WH: contributed to the methodological description, contributed to manuscript revisions, and reviewed the programming code and description thereof. JK: coordinated laboratory analyses, and including reference materials. AD, RB, and EJ: contributed to manuscript revisions. All authors contributed to the article and approved the submitted version.

## Footnotes

^1^With a span of 0.25 a quarter of all points is used at each given day, which have a weight based on distance as (1−(distance/distance_max_)^3^)^3^. This gives points closest to the day of interest a much higher weight than more distant points.
