# Supplementary material for: PCR standard curve quantification in an extensive wastewater surveillance program: results from the Dutch SARS-CoV-2 wastewater surveillance
Source: Front Public Health. 2023 Nov 2;11:1141494. doi: 10.3389/fpubh.2023.1141494 (PMC10652756; doi:10.3389/fpubh.2023.1141494)
Supplement: Supplementary file 1 [file Data_Sheet_1.docx]

Supplementary Material

PCR standard curve quantification in an extensive wastewater surveillance program: Results from the Dutch SARS-CoV-2 Wastewater Surveillance

Erwin Nagelkerke^1*^, Jaap M. Koelewijn^1^, Eline F. de Jonge^1^, Jannetje Kooij^1^, Anne-Merel R. van der Drift^1^, Wouter A. Hetebrij^1^, Consortium NRS^1^, Ruud F. H. J. van der Beek^1^, Willemijn J. Lodder^1^

^1^ Centre for Zoonoses and Environmental Microbiology, National Institute for Public Health and the Environment, the Netherlands

*** Correspondence:**Corresponding Author, erwin.nagelkerke@rivm.nl

# Supplement

## S1: CDC N1-N2-N3 G-block Sequence

**TAATACGACTCACTATAGGGAGACCACAAC**GTGTTGTTTTAGATTTCATCTAAACGAACAAACTAAAATGTCTGATAATGGACCCCAAAATCAGCGAAATGCACCCCGCATTACGTTTGGTGGACCCTCAGATTCAACTGGCAGTAACCAGAATGGAGAACGCAGTGGGGCGCGATCAAAACAACGTCGGCCCCAAGGTTTACAAACCCAAGGAAATTTTGGGGACCAGGAACTAATCAGACAAGGAACTGATTACAAACATTGGCCGCAAATTGCACAATTTGCCCCCAGCGCTTCAGCGTTCTTCGGAATGTCGCGCATTGGCATGGAAGTCACACCTTCGGGAACGTGGTTGACCTACACAGGTGCGGACTTCCCTATGGTGCTAACAAAGACGGCATCATATGGGTTGCAACTGAGGGAGCCTTGAATACACCAAAAGATCACATTGGCACCCGCAATCCTGCTAACAATGCTGCAATCGTGCTACAACTTCCTCAAGGAACAACATTGCCAAAAGGCTTCTACGCAGAAGGGAGCA

The T7 promoter is shown in bold, and underlined are the N1, N2, and N3 primer and probe locations, respectively.

## S2: QIAquant Mean Differences

To test mean differences between QIAquant machines an OLS regression is performed with all the machines entered through dummy coding. The model F-test for this regression analysis reduces to an ANOVA omnibus test of means differences, but with the added benefits that the regression specification allows inclusion of the interaction effect of machine by slope, and directly provides the t-tests for pairwise means difference testing.

To reduce the effect of possible time-by-machine interactions, the daily mean of both the intercept and slope are obtained over all available curves on that day and subtracted from the individual machine slopes and intercepts. The coefficients presented in table S1 then compare the average deviation from the daily mean per machine to that of reference machine (1). To test the full range of possibilities, the interaction effects of machine by slope were also tested, and despite some individual pairwise comparisons reaching statistical significance at the 0.050 level, the explained variance of including interactions on top of a main effect equal 0.41% and 0.83% for N1 and N2.

**Table S1**. Means differences in slope and intercept coefficients per instrument used for the RT-qPCR analyses (QIAquant). Data is mean centered, reference is the instrument closest to the mean intercept.

|  | N1 | | | |  | N2 | | | |
| --- | --- | --- | --- | --- | --- | --- | --- | --- | --- |
|  | Intercept | | Slope | |  | Intercept | | Slope | |
|  | B | SE | B | SE |  | B | SE | B | SE |
| Ref. (1) | .001 | .035 | -.003 | .007 |  | -.014 | .034 | .005 | .006 |
| (2) | -.028 | .045 | .013 | .008 |  | -.025 | .048 | .007 | .008 |
| (3) | .032 | .077 | .009 | .014 |  | -.018 | .082 | .035 | .014 |
| (4) | -.006 | .045 | -.004 | .008 |  | -.079 | .050 | .010 | .009 |
| (5) | .101* | .046 | -.010 | .009 |  | .075 | .050 | -.013 | .009 |
| (6) | -.024 | .062 | -.001 | .012 |  | -.145 | .070 | .023 | .012 |
| (7) | -.037 | .046 | .006 | .009 |  | -.081 | .054 | .011 | .009 |
| (8) | -.025 | .048 | .010 | .009 |  | -.204 | .052 | .017 | .009 |
| (9) | -.056 | .071 | .014 | .013 |  | -.088 | .080 | .036 | .014 |
| R^2^ \| F-value | .004 | 1.958 | .004 | 1.739 |  | .011 | 4.239 | .009 | 3.203 |

** = p-value < .010 | * = p-value < .050

## S3: Curve parameters N2


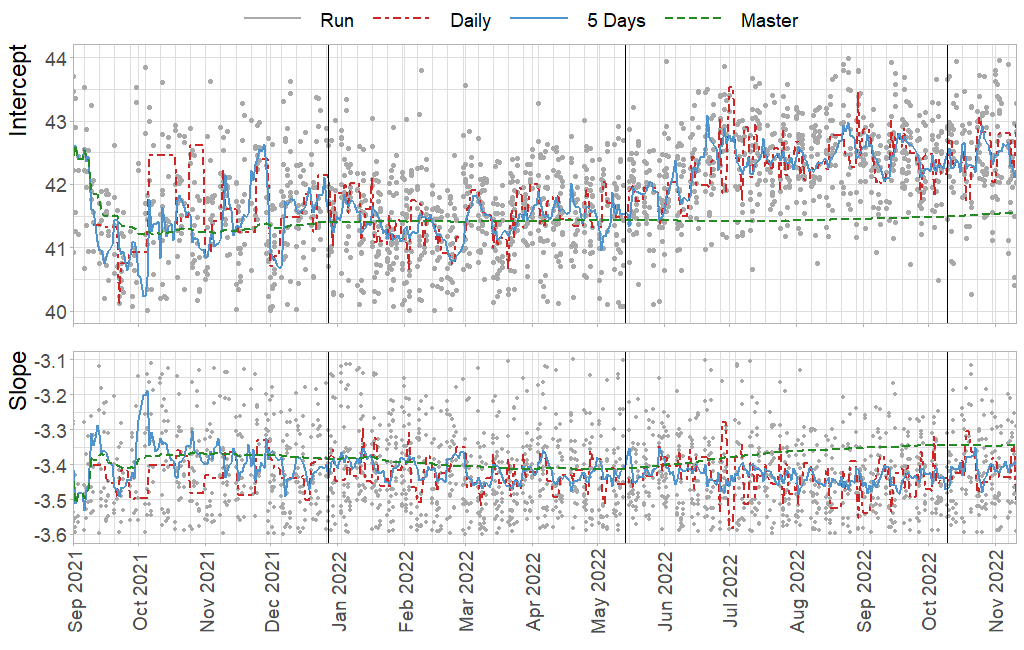


Figure S1. Estimated standard parameters for the N2 target, based on estimation per run (grey), on all daily observations of standard dilutions (red), on all observations within a five day rolling window (blue) and on all cumulative data up to that point (green). Vertical lines indicate a new preparation from the CDC assay.
